# LONGITUDINAL DYADIC ASSOCIATIONS BETWEEN LONELINESS AND COGNITION AMONG OLDER COUPLES IN THE UNITED STATES

**DOI:** 10.1093/geroni/igae098.1191

**Published:** 2024-12-31

**Authors:** Jeffrey Stokes, Anyah Prasad, Adrita Barooah, Elisabeth Stam

**Affiliations:** University of Massachusetts Boston, Boston, Massachusetts, United States; University of Massachusetts Boston, Boston, Massachusetts, United States; University of Massachusetts Boston, Boston, Massachusetts, United States; University of Massachusetts Boston, Boston, Massachusetts, United States

## Abstract

**Objectives:**

Loneliness is associated with diminished health and cognition for older individuals. However, little research has examined dyadic loneliness – i.e., loneliness of both partners in a relationship – and its potential consequences for cognitive functioning among both spouses, nor whether one partner’s cognition may impact both partners’ loneliness over time.

**Methods:**

We analyze 3-wave dyadic Health and Retirement Study data (2010-2020; N=1,061 dyads) to determine (a) whether loneliness predicts participants’ own and/or their partners’ episodic memory and verbal fluency over 8 years, and (b) whether cognitive functioning predicts older spouses’ own or their partners’ loneliness over the same period.

**Results:**

Loneliness predicted participants’ own and their partners’ loneliness at follow-up, at both timepoints. Loneliness was also associated with own episodic memory at follow-up, but not with verbal fluency. Episodic memory and verbal fluency predicted one another over time. Neither episodic memory nor verbal fluency predicted loneliness at follow-up. Significant dyadic mediation was established such that Time 1 loneliness was linked with partner’s Time 3 episodic memory via that partner’s Time 2 loneliness.

**Discussion:**

Lonelier older adults displayed worse trajectories of episodic memory over time, yet poor memory did not precede changes to loneliness. Further, having a lonely partner was linked with poorer episodic memory 8 years later, indicating that both one’s own and – to a lesser extent – a partner’s emotional well-being may be consequential for maintaining cognitive functioning with age. Associations were more clearly established with episodic memory than with verbal fluency, suggesting domain-specific effects of loneliness.

